# Clinicopathological and ultrasound characteristics of breast cancer in *BRCA1* and *BRCA2* mutation carriers

**DOI:** 10.1007/s10396-023-01296-w

**Published:** 2023-03-11

**Authors:** Kengo Ikejima, Sayuri Tokioka, Kazuyo Yagishita, Yuka Kajiura, Naoki Kanomata, Hideko Yamauchi, Yasuyuki Kurihara, Hiroko Tsunoda

**Affiliations:** 1 Department of Radiology, St. Luke’s International Hospital, 9-1 Akashi-Cho, Chuo-Ku, Tokyo, 104-8560 Japan; 2 Sendai Cardiovascular Center, 1-6-12 Izumichuo, Izumi-Ku, Sendai, Miyagi 981-3133 Japan; 3 Department of Breast Surgical Oncology, St. Luke’s International Hospital, 9-1 Akashi-Cho, Chuo-Ku, Tokyo, 104-8560 Japan; 4 Department of Pathology, St. Luke’s International Hospital, 9-1 Akashi-Cho, Chuo-Ku, Tokyo, 104-8560 Japan

**Keywords:** *BRCA* mutation, Ultrasound, Breast cancer, Tumor morphology, Retrospective study

## Abstract

**Purpose:**

*BRCA1* and *BRCA2* tumors exhibit different characteristics. This study aimed to assess and compare the ultrasound findings and pathologic features of *BRCA1* and *BRCA2* breast cancers. To our knowledge, this is the first study to examine the mass formation, vascularity, and elasticity in breast cancers of *BRCA*-positive Japanese women.

**Methods:**

We identified patients with breast cancer harboring *BRCA1* or *BRCA2* mutations. After excluding patients who underwent chemotherapy or surgery before the ultrasound, we evaluated 89 cancers in *BRCA1*-positive and 83 in *BRCA2*-positive patients. The ultrasound images were reviewed by three radiologists in consensus. Imaging features, including vascularity and elasticity, were assessed. Pathological data, including tumor subtypes, were reviewed.

**Results:**

Significant differences in tumor morphology, peripheral features, posterior echoes, echogenic foci, and vascularity were observed between *BRCA1* and *BRCA2* tumors. *BRCA1* breast cancers tended to be posteriorly accentuating and hypervascular. In contrast, *BRCA2* tumors were less likely to form masses. In cases where a tumor formed a mass, it tended to show posterior attenuation, indistinct margins, and echogenic foci. In pathological comparisons, *BRCA1* cancers tended to be triple-negative subtypes. In contrast, *BRCA2* cancers tended to be luminal or luminal-human epidermal growth factor receptor 2 subtypes.

**Conclusion:**

In the surveillance of *BRCA* mutation carriers, radiologists should be aware that the morphological differences between tumors are quite different between *BRCA1* and *BRCA2* patients.

**Supplementary Information:**

The online version contains supplementary material available at 10.1007/s10396-023-01296-w.

## Introduction

*BRCA* mutations increase the risk of breast cancer; so, in *BRCA* mutation carriers, it is essential to perform different surveillance compared with women of average breast cancer risk to detect and treat breast cancers in the early stages. Since mammography is not suitable for breast cancer surveillance in *BRCA* patients, magnetic resonance imaging (MRI) is considered the standard surveillance modality for *BRCA* carriers [1].

*BRCA1* and *BRCA2* tumors have different characteristics. The triple-negative (TN) subtype is common in *BRCA1* breast cancer. In contrast, the luminal subtype is a common subtype of breast cancer in *BRCA2* mutation carriers, which has a similar frequency to non-*BRCA* breast cancers [2]. We hypothesized that due to their histological differences, *BRCA1* and *BRCA2* breast cancers demonstrate different imaging characteristics on ultrasound. Pathologic and ultrasound features of *BRCA1* and *BRCA2* breast cancers were previously assessed, although several essential variables, such as vascularity or elasticity, remain unknown [3]. Also, there are few studies assessing imaging features of *BRCA* breast cancers in the Japanese population.

Therefore, we aimed to assess and compare ultrasound image findings, including vascularity and elasticity, and pathologic features of *BRCA1* and *BRCA2* breast cancers in Japanese women based on the categories of the Japan Association of Breast and Thyroid Sonology (JABTS) guidelines. Our primary endpoint was to assess and compare the differences in ultrasonographic features between *BRCA1* and *BRCA2* breast cancers. Our secondary endpoint was the difference in clinicopathological characteristics between them.

## Materials and methods

### Study population

**Fig. 1:**
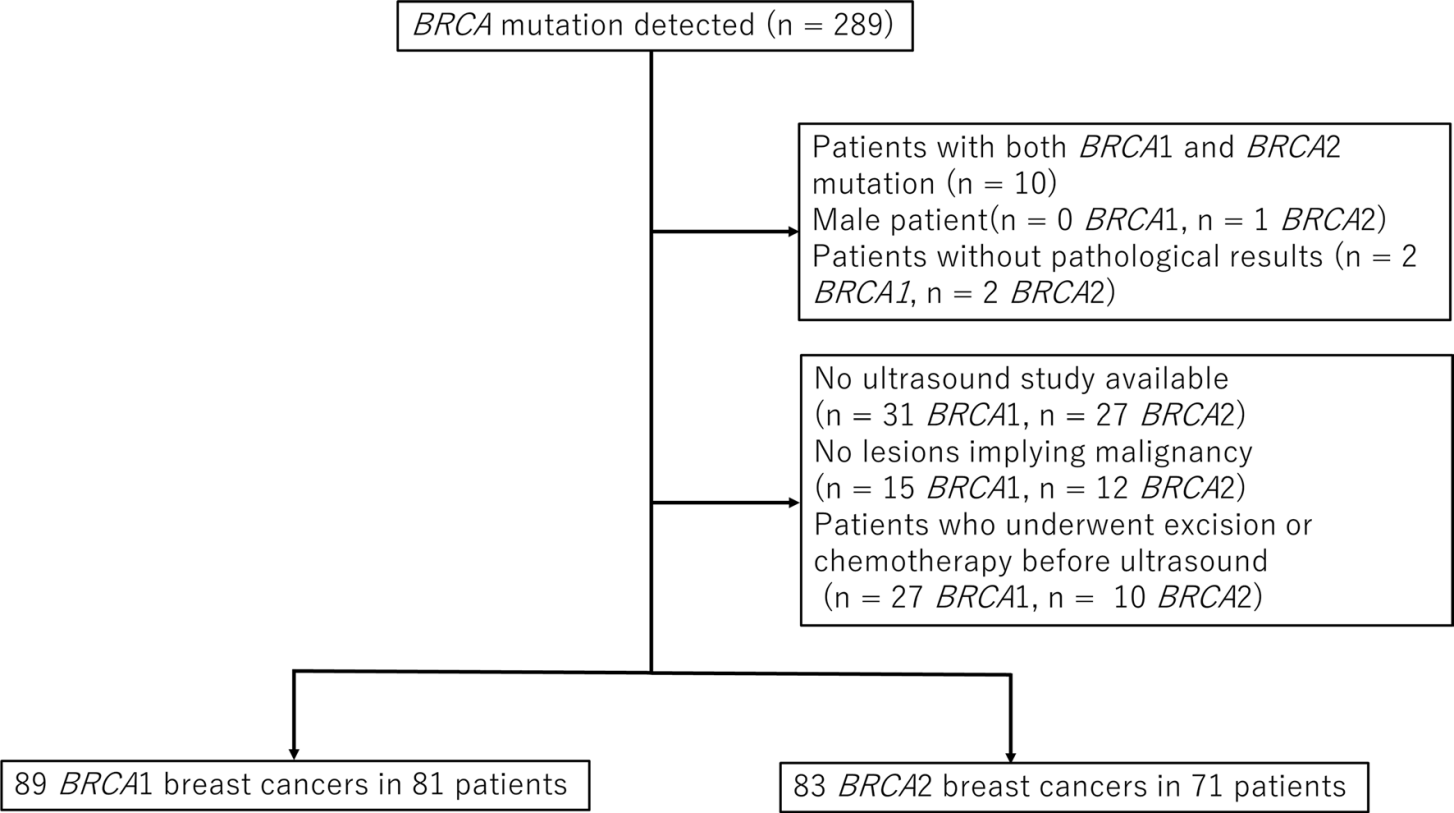
Patient selection flowchart

A flowchart of the patient selection process is shown in Fig. 1.

This retrospective cohort study was conducted at St. Luke’s Hospital. Patients with breast cancer harboring *BRCA1* or *BRCA2* mutations between July 2003 and December 2020 were included in the study.

At our institution, we recommend *BRCA* tests for breast cancer patients who have any of the following characteristics:

- Under 45 years old
- TN cancer and under 60 years old
- Bilateral breast cancer
- Male breast cancer
- Family history of breast cancer

The following patients were excluded: patients who underwent preoperative chemotherapy before the ultrasound, patients without lesions that implied malignancy, patients with both *BRCA1* and *BRCA2* mutations, patients without pathological results, and male patients.

Our study was approved by the institutional review board of our hospital, and the requirement for informed consent was waived due to the retrospective nature of the study (Research number: 20-R238).

### Imaging technique

Breast sonography was performed by radiologists and radiology technologists, and all images were assessed by radiologists specializing in breast imaging. Breast sonography was performed at our hospital using one of the following devices: HDI 5000 (Philips Medical Systems, Best, the Netherlands), LOGIQ7 (GE Healthcare, Chicago, IL, USA), EUB-7500 (FUJIFILM Healthcare Corporation, formerly called Hitachi Corporation, Tokyo, Japan), Aplio XG (Toshiba Medical Systems, Tochigi, Japan), HIVISION Preirus (Hitachi Healthcare Corporation, Tokyo, Japan), LOGIC E9 (GE Healthcare), and Aplio i800 (Canon Medical Systems Corporation, Tochigi, Japan). The details of the ultrasound equipment utilized for five patients could not be verified because the ultrasound examinations were performed at the referring hospital. The images were then reviewed and evaluated. Ultrasound findings were checked for B-mode, elastography, and flow imaging (color Doppler image) information.

### Data analysis

We assessed and classified the image morphologies based on shape, margin, homogeneity, echo level, vascularity, and elasticity. Three radiologists (one with 4 years of experience in general radiology, one with 2 years of experience in breast radiology, and one with 30 years of experience in breast radiology) reviewed the ultrasound images and reports. Findings were recorded after reaching a consensus. If the assessment was different, we reviewed and discussed the images together and reached a consensus.

Imaging features were assessed based on the JABTS guidelines. Some terms in the JABTS guidelines are different from the terms in the ACR BIRADS 2013 Ultrasound (Supplementary information). The most significant difference is that the JABTS guidelines classify lesions that cannot be strictly traced as masses as “non-mass abnormalities, ” a category separate from masses. Regarding the classification of vascularity, we defined “high” when tumors were hypervascular and “low” when tumors were hypovascular or avascular. Elasticity was assessed using strain elastography. We used the scoring system introduced by Itoh et al. [4].

The imaging features analyzed in this study are shown in Tables 1 and 2. On B-mode imaging, we classified the tumor morphology as “mass only,” “non-mass abnormalities only,” and “mass with non-mass abnormalities.” We defined “non-mass abnormalities” as tumors in which a definite contour could not be identified. Shape, depth-width ratio, margin, echogenic halo, homogeneity, echo level, and posterior echoes were evaluated in lesions with mass abnormality only and in those with mass and non-mass abnormalities (*BRCA1,* *n* = 85; *BRCA2,* *n* = 62). The associated findings were evaluated in cases with non-mass abnormalities only and in those with mass and non-mass abnormalities (*BRCA1,* *n* = 56; *BRCA2,* *n* = 65). Echogenic foci, vascularity, and elasticity were evaluated in all cases. While counting the number of cases with echogenic foci, we did not distinguish whether the echogenic foci had hypoechoic areas or not. Moreover, we reviewed pathological data, including tumor histological classification, nuclear grade, and tumor subtype.

**Table 1:** Imaging features analyzed in this study

Tumor morphology	
	Mass abnormality only Non-mass abnormalities only Mass abnormality with non-mass abnormalities
Findings (of mass abnormality)	
Shape	Round/Oval/Lobulated/Polygonal/Irregular
Depth-width ratio	< 0.5, 0.5–0.7, > 0.7
Margin	Circumscribed/Well-defined and Rough/Indistinct
Echogenic halo	Present or Absent
Homogeneity	Homogeneous or Heterogeneous
Echo level	Hyperechoic/Isoechoic/Hypoechoic/Anechoic
Posterior echoes	Accentuating/Not changing/Attenuating/Shadowing
Findings (of non-mass abnormalities)	
Abnormality of the ducts	Present or Absent
Hypoechoic area in the mammary gland	Present or Absent
Architectural distortion	Present or Absent
Multiple small cysts	Present or Absent
Echogenic foci (in all abnormalities)	Present or Absent
Vascularity (in all abnormalities)	High/Low/Unknown
Elasticity (in all abnormalities)	Score 1, 2, 3, 4, 5/unknown

**Table 2:** Vascularity and elasticity scores

Vascularity	
High	Hypervascular
Low	Hypovascular or avascular
Elasticity scores	
1	Even strain for the entire hypoechoic lesion
2	Strain in most of the hypoechoic lesion, with some areas of no strain
3	Strain at the periphery of the hypoechoic lesion, with sparing of the center of the lesion
4	No strain in the entire hypoechoic lesion
5	No strain in the entire hypoechoic lesion or in the surrounding area

Breast cancer subtypes were categorized into the following four groups: hormone receptor-positive and human epidermal growth factor receptor 2 (HER-2) negative (luminal subtype), hormone receptor-positive and HER-2 positive (luminal-HER-2 subtype), hormone receptor-negative and HER-2 positive (HER-2 subtype), and hormone receptor-negative and HER-2 negative (TN subtype). Luminal tissue was classified as positive when the Allred score of either the estrogen receptor or progesterone receptor was higher than 2. HER-2 was classified as positive if the immunohistochemistry score was 3 or if the immunohistochemistry score was 2 and the fluorescence in situ hybridization test was positive.

### Statistical analysis

Missing data from some patients were excluded from the analysis. Since some patients had two or more breast cancers, statistical analysis was performed based on the number of cancers. Data are presented as the mean ± standard deviation or median (range) for continuous variables, such as age and Ki-67, and as numbers (percentages) for categorical variables. We used the *t*-test and Mann–Whitney *U* test for continuous variables and the chi-squared and Fisher’s exact tests for categorical variables to compare the characteristics of participants with *BRCA1* and *BRCA2*. Continuous variables were verified by Shapiro–Wilk statistics. A *p* value < 0.05 indicated a statistically significant difference between groups. If there were statistically significant differences for multiple comparisons, we performed Bonferroni correction as a post hoc analysis. Bonferroni correction is used to compare multiple comparisons. In multiple comparisons, the likelihood of incorrectly rejecting a null hypothesis (type I error) increases. In Bonferroni correction, the alpha value is divided by the number of comparisons to prevent a type I error.

All statistical analyses were performed using EZR (Saitama Medical Center, Jichi Medical University, Saitama, Japan), a graphical user interface for R (The R Foundation for Statistical Computing, Vienna, Austria) [5].

## Results

A total of 289 patients (156 *BRCA1* positive, 123 *BRCA2* positive, and 10 who were both *BRCA1* and *BRCA2* positive) were identified. Seventy-three *BRCA1*-positive patients and 49 *BRCA2*-positive patients were excluded due to the absence of ultrasound images before chemotherapy or surgery. After excluding ineligible patients, a total of 89 cancers in 81 patients with *BRCA1*-positive mutations and 83 cancers in 71 patients with *BRCA2*-positive mutations were included.

Table 3 summarizes the clinicopathological characteristics of the patients. Continuous variables such as age and Ki-67 were refuted, and the Mann–Whitney *U* test was used for their analysis. Since there were significant differences in tumor type, tumor subtype, and nuclear grade, we performed Bonferroni corrections. The median patient age was 40 (28–70) years in *BRCA1* mutation carriers and 42 (28–73) years in *BRCA2* mutation carriers. Thus, there was no significant difference in patient age between the two groups (*p* = 0.288). However, there was a significant difference in the tumor histological classification and subtype (*p* < 0.001). After Bonferroni correction, *BRCA1* tumors were most frequently associated with invasive ductal carcinoma, whereas *BRCA2* tumors were more frequently associated with ductal carcinoma in situ (*p* < 0.001). *BRCA1* tumors were associated with the TN subtype (*p* < 0.001). In contrast, the most common type of *BRCA2* breast cancer was luminal-type breast cancer, and the luminal-HER2 type was more frequent in *BRCA2* than *BRCA1* tumors (*p* < 0.001). The nuclear grade (*p* < 0.001) and Ki-67 status (*p* < 0.001) of *BRCA1* tumors were significantly higher than those of *BRCA2* tumors.

**Table 3:** Clinicopathological characteristics of breast cancer in the study participants

Variable	*BRCA1* (*n* = 89)	*BRCA2* (*n* = 83)	*p*
Patient age (y), median (range)	40 (28–70)	42 (28–73)	0.288
Tumor size (cm)			0.54
< 2	42 (47.2)	27 (32.5)	
> 2	47 (52.8)	56 (67.5)	
Tumor type			< 0.001
Invasive ductal carcinoma NOS	82 (92.1)	60 (72.3)	
Invasive lobular carcinoma	0 (0)	1 (1.2)	
Medullary carcinoma	3 (3.4)	0 (0)	
Ductal carcinoma in situ	4 (4.5)	21 (35.3)	
Paget disease	0 (0)	1 (1.2)	
Tumor subtype			< 0.001
Luminal	37 (41.6)	54 (65.6)	
HER2	4 (4.5)	3 (3.6)	
Luminal HER2	0 (0)	8 (9.6)	
Triple-negative	46 (51.7)	10 (12.0)	
Unknown	2 (2.2)*	8 (9.6)**	
Nuclear grade			< 0.001
1	9 (10.1)	22 (26.5)	
2	22 (24.7)	35 (42.2)	
3	58 (65.2)	26 (31.3)	
Ki-67 status, median (range)	65 (18–100)	42 (7–100)	< 0.001
Unknown	10	12	

*NOS* not otherwise specified, *HER2* human epidermal growth factor receptor 2

^*^Pathological evaluations were performed at the referring hospital

^**^All eight cases were HER2 immunohistochemistry score 2 ductal carcinoma in situ. At our hospital, the HER2 FISH (fluorescence in situ hybridization) test is not performed for ductal carcinoma in situ cases

**Fig. 2:**
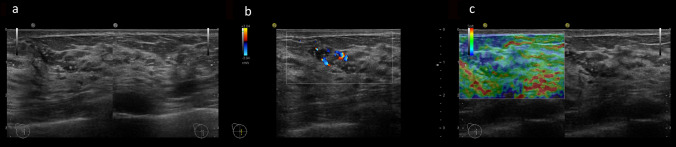
A 46-year-old woman with a *BRCA2* mutation and ductal carcinoma in situ (luminal-HER2 subtype). Breast ultrasound demonstrates a hypoechoic area with calcifications. We defined this morphology as no mass. The hypoechoic area shows high vascularity and score 5 elasticity (no strain in the entire lesion or surrounding area). *HER2* human epidermal growth factor receptor 2

Table 4 summarizes the imaging characteristics of the patients. Since there were significant differences in tumor morphology, margin, and posterior features, we performed Bonferroni corrections. There was a significant difference in tumor morphology between *BRCA1* and *BRCA2* tumors (*p* < 0.001). After Bonferroni correction, *BRCA2* tumors were significantly more likely to show non-mass abnormalities only (*p* < 0.001 vs. mass abnormality only, *p* = 0.002 vs. mass abnormality with no mass abnormalities) (Fig. 2).

**Table 4:** Imaging features of *BRCA1* and *BRCA2* breast cancers

Variable	*BRCA1* (n = 89)	*BRCA2* (n = 83)	*p*
Tumor morphology			< 0.001
Mass abnormality only	33 (37.1)	18 (21.7)	
Non-mass abnormalities only	4 (4.5)	21 (25.3)	
Mass abnormality with non-mass abnormalities	52 (58.4)	44 (53.0)	
Mass			
Shape			0.27
Round	12 (14.1)	8 (12.9)	
Oval	16 (18.8)	5 (8.1)	
Irregular	27 (31.8)	29 (46.8)	
Polygonal	6 (7.1)	4 (6.5)	
Lobulated	24 (28.2)	16 (25.8)	
Depth-width ratio			0.63
< 0.5	4 (4.7)	1 (1.6)	
0.5–0.7	12 (14.1)	8 (12.9)	
> 0.7	69 (81.2)	53 (85.5)	
Margin			0.035
Circumscribed	13 (15.3)	5 (8.1)	
Well-defined and rough	47 (55.3)	26 (41.9)	
Indistinct	25 (29.4)	31 (50.0)	
Echogenic halo			0.43
Present	17 (20.0)	16 (25.8)	
Absent	68 (80.0)	46 (74.2)	
Homogeneity			0.44
Homogeneous	18 (21.2)	17 (27.4)	
Heterogeneous	67 (78.8)	45 (72.6)	
Echo level			0.12
Hyperechoic	1 (1.2)	2 (3.2)	
Isoechoic	1 (1.2)	2 (3.2)	
Hypoechoic	78 (91.8)	58 (93.5)	
Anechoic	5 (5.9)	0 (0)	
Posterior features			< 0.001
Accentuating	60 (70.6)	21 (33.9)	
Not changing	18 (21.2)	25 (40.3)	
Attenuating	7 (8.2)	16 (25.8)	
Shadowing	0 (0)	0 (0)	
Echogenic foci	28 (31.5)	39 (47.0)	0.043
Non-mass abnormalities			
Abnormality of the ducts	10 (16.4)	16 (21.1)	0.20
Hypoechoic area in the mammary gland	51 (83.6)	59 (77.6)	0.080
Architectural distortion	0 (0)	1 (1.3)	0.48
Multiple small cysts	0 (0)	0 (0)	
Vascularity			0.031
High	68 (76.4)	50 (60.2)	
Low	20 (22.5)	31 (37.3)	
Unknown	1 (1.1)	2 (2.4)	
Elasticity (score)			0.25
1	1 (1.5)	2 (3.3)	
2	4 (5.9)	2 (3.3)	
3	8 (11.8)	9 (14.8)	
4	36 (52.9)	22 (36.1)	
5	19 (27.9)	26 (42.6)	
Unknown	21	22

**Fig. 3:**
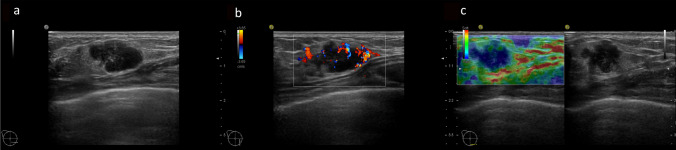
A 38-year-old woman with a *BRCA1* mutation and high-grade invasive ductal carcinoma (triple-negative subtype). Breast ultrasound demonstrates a circumscribed polygonal mass without calcification. This tumor has high vascularity and score 4 elasticity (no strain in the entire lesion)

Among the cases with mass formation, there were no significant differences in shape (*p* = 0.27), depth-width ratio (*p* = 0.63), presence or absence of echogenic halo (*p* = 0.43), homogeneity (*p* = 0.44), or level of internal echoes (*p* = 0.12). There was a significant difference in the peripheral features (*p* = 0.035). *BRCA2* tumors were more likely to show indistinct margins compared with *BRCA1* tumors. Additionally, there was a significant difference in the posterior acoustic enhancement; after Bonferroni correction, *BRCA1* tumors significantly tended to show accentuating posterior echoes (*p* = 0.001 vs. attenuating, *p* = 0.002 vs. not changing) (Fig. 3).

Additionally, there was no significant difference in the features of non-mass abnormalities, including abnormality of the ducts (*p* = 0.20), hypoechoic areas in the mammary gland (*p* = 0.080), architectural distortion (*p* = 0.48), and multiple small cysts (no cases).

With regard to flow imaging, *BRCA1* tumors tended to show higher vascularity than *BRCA2* tumors (*p* = 0.031). On elastography, *BRCA1* tended to have a score of 4 more often. However, the number of patients with a score of 4 or higher, which is considered indicative of malignancy, was almost the same, at 55/68 (80.9%) for *BRCA1* and 48/61 (78.7%) for *BRCA2*.

We detected eight cancer cases with the luminal-HER2 subtype in *BRCA2* tumors, but none in *BRCA1*. Of these luminal-HER2 tumors, five cases did not form masses, four of which had echogenic foci. Moreover, *BRCA2* tumors were significantly more likely than *BRCA1* tumors to show echogenic foci (*p* = 0.043).

## Discussion

To our knowledge, this is the first study summarizing ultrasound findings of breast cancer in *BRCA*-positive patients in the Japanese population. *BRCA* is known to be a high-risk factor for breast cancer, and the frequency of encountering *BRCA*-positive patients in daily clinical practice has increased since *BRCA* testing for some individuals started being covered by insurance in Japan. The indication of *BRCA* testing in Japan includes patients with breast cancer who have any of the following characteristics: < 45 years, TN cancer and < 60 years, two or more breast cancers, male breast cancer, family history of breast cancer within the third-degree relatives. Therefore, it is vital to understand the imaging characteristics of *BRCA*-positive breast cancer cases.

There is an international consensus that MRI is useful for the surveillance of *BRCA*-positive patients, and many articles on the imaging characteristics of MRI have already been published [1, 6–8]. However, few papers have been published on the imaging features of ultrasound, particularly color Doppler imaging or elastography, especially in Japanese women.

The Japanese guidelines were used in this study because some of the breast ultrasound images could not be described as masses; the Japanese guidelines treat these as “non-mass abnormalities.” Non-mass abnormalities are not introduced in the ACR BIRADS 2013 Ultrasound (Supplementary information). The history of breast ultrasound in Japan is long; thus, the Japanese guidelines are considered to be the default in routine clinical settings.

In the current study, we found that *BRCA1* tumors were more likely to show distinct or well-defined and rough margins, accentuating posterior echoes, and high vascularity. *BRCA2* tumors were less likely to form masses and had more echogenic foci. *BRCA1*-associated breast cancers are known to frequently present with benign features, such as round shape, circumscribed margins, and a homogeneous internal structure [9]. Ha et al. compared the clinicopathological and imaging findings of *BRCA1* and *BRCA2* mutations. They revealed that *BRCA1* tumors exhibited posterior acoustic enhancement (p < 0.001) [3], which is in agreement with our findings. Regarding tumor subtypes, *BRCA1* breast cancers were associated with a TN subtype, whereas *BRCA2* breast cancers were associated with a luminal subtype. These results are consistent with those of a previous study [2]. TN breast cancers are known to be associated with an increased likelihood of distant recurrence and death [10]. The TN breast cancer subtype tends to be a circumscribed tumor with posterior acoustic enhancement [11, 12], which sometimes mimics benign lesions like fibroadenoma. However, in our study, *BRCA1* tumors tended to have well-defined and rough margins, high depth-width ratio, and hypervascularization, which are uncommon features of benign tumors like fibroadenoma. If these findings are assessed carefully, we could avoid the misidentification of *BRCA1* breast cancers. In contrast, *BRCA2* tumors tend to be luminal subtypes, which tend to be non-circumscribed tumors with posterior attenuation or shadowing [12]. These morphological differences suggest that patients with *BRCA1* and *BRCA2* mutations should be assessed differently.

The rates of echogenic foci, usually representing microcalcifications, and non-mass abnormalities were significantly higher in *BRCA2* than in *BRCA1* patients. This result may also be attributed to tumor histological differences, where ductal carcinoma in situ is more common in *BRCA2*. NCC guidelines recommend that surveillance of breast cancer should be conducted with both mammography and MRI in *BRCA* patients. Although we did not compare the findings of mammography between *BRCA1* and *BRCA2* patients, the higher rate of echogenic foci in *BRCA2* cancers might suggest the importance of mammography in *BRCA2* patients. However, not even half of the *BRCA2* tumors had echogenic foci, indicating that ultrasound could be a good option in situations where MRI cannot be used. We assessed several important variables in our study, including vascularity and elasticity. We hypothesized that *BRCA1* tumors have score 4 elasticity (no strain in the entire lesion) rather than scores 1, 2, 3, or 5 because TN subtype (which is common in *BRCA1* cancers) tumors are likely to have well-circumscribed margins. Our results showed that *BRCA1* breast cancers tended to demonstrate score 4 elasticity; however, there was no significant difference between *BRCA1* and *BRCA2* breast cancers. This result might be due to the low number of cancers assessed or the excessively granular subdivision of the scale. *BRCA1* tumors were significantly hypervascular compared with *BRCA2* tumors. This result reflects the TN subtype tendency of *BRCA1* breast cancer.

This study had several limitations. First, it was a retrospective study where radiologists gathered data regarding the presence of cancer. Second, there were several missing data points in some patients, especially in the vascularity and elasticity scores. Third, although some cases were reviewed by several radiologists, most cases were assessed and reviewed by a single radiologist. Finally, this study was performed at a single hospital. A large multicenter study is required to verify our findings.

## Conclusion

Our results suggest that *BRCA1* breast cancers tend to be masses, posteriorly accentuating, and hypervascular. In contrast, *BRCA2* tumors are less likely to form masses. In cases where a tumor forms a mass, it tends to show posterior attenuation, indistinct margins, and echogenic foci. In the surveillance of *BRCA* mutation carriers, radiologists should be aware that the morphological differences between tumors are quite different between *BRCA1* and *BRCA2* patients.

## Supplementary Information

Below is the link to the electronic supplementary material.

Supplementary file1 (DOCX 29 KB)

## Data Availability

The datasets analyzed during the current study are available from the corresponding author on reasonable request.
