# Correction: Clinicopathological and ultrasound characteristics of breast cancer in *BRCA1* and *BRCA2* mutation carriers

**DOI:** 10.1007/s10396-023-01325-8

**Published:** 2023-05-25

**Authors:** Kengo Ikejima, Sayuri Tokioka, Kazuyo Yagishita, Yuka Kajiura, Naoki Kanomata, Hideko Yamauchi, Yasuyuki Kurihara, Hiroko Tsunoda

**Affiliations:** 1 Department of Radiology, St. Luke’s International Hospital, 9-1 Akashi-Cho, Chuo-Ku, Tokyo, 104-8560 Japan; 2 Sendai Cardiovascular Center, 1-6-12 Izumichuo, Izumi-Ku, Sendai, Miyagi 981-3133 Japan; 3 Department of Breast Surgical Oncology, St. Luke’s International Hospital, 9-1 Akashi-Cho, Chuo-Ku, Tokyo, 104-8560 Japan; 4 Department of Pathology, St. Luke’s International Hospital, 9-1 Akashi-Cho, Chuo-Ku, Tokyo, 104-8560 Japan

**Correction: Journal of Medical Ultrasonics (2023) 50:213–220** 10.1007/s10396-023-01296-w

The article “Clinicopathological and ultrasound characteristics of breast cancer in BRCA1 and BRCA2 mutation carriers”, written by Kengo Ikejima, Sayuri Tokioka, Kazuyo Yagishita, Yuka Kajiura, Naoki Kanomata, Hideko Yamauchi, Yasuyuki Kurihara and Hiroko Tsunoda, was originally published Online First without Open Access. After publication in volume 50, issue 2, pages 213–220 the author decided to opt for Open Choice and to make the article an Open Access publication. Therefore, the copyright of the article has been changed to © The Author(s) 2023 and the article is forthwith distributed under the terms of the Creative Commons Attribution 4.0 International License, which permits use, sharing, adaptation, distribution and reproduction in any medium or format, as long as you give appropriate credit to the original author(s) and the source, provide a link to the Creative Commons licence, and indicate if changes were made. The images or other third party material in this article are included in the article’s Creative Commons licence, unless indicated otherwise in a credit line to the material. If material is not included in the article’s Creative Commons licence and your intended use is not permitted by statutory regulation or exceeds the permitted use, you will need to obtain permission directly from the copyright holder. To view a copy of this licence, visit http://creativecommons.org/licenses/by/4.0.

