# Supplementary material for: Clinicopathological and ultrasound characteristics of breast cancer in BRCA1 and BRCA2 mutation carriers
Source: J Med Ultrason (2001). 2023 Mar 11;50(2):213–20. doi: 10.1007/s10396-023-01296-w (PMC10202977; doi:10.1007/s10396-023-01296-w)
Supplement: Supplementary file 1 — Supplementary file1 (DOCX 29 KB) [file 10396_2023_1296_MOESM1_ESM.docx]

Ref. Findings in JABTS guidelines and ACR BIRADS 2013 Ultrasound

|  |  | JABTS guidelines | ACR BIRADS 2013 |
| --- | --- | --- | --- |
| Masses | Shape | Round  Oval  Lobulated  Polygonal  Irregular | Oval  Round  Irregular |
|  | Orientation  Depth-width ratio | <0.5  0.5–0.7  >0.7 | Parallel  Not parallel |
|  | Margin | Well-defined and smooth  (circumscribed)  Well-defined and rough  Indistinct | Circumscribed  Not circumscribed   1. Indistinct 2. Angular 3. Microlobulated 4. Spiculated |
|  | Echogenic halo | Present or Absent |  |
|  | Echo pattern  Echo level | Cystic  Mixed  Solid  Fluid-fluid level  Hyperechoic, High  Isoechoic, Equal  Hypoechoic, Low  Anechoic, Absent | Anechoic  Hyperechoic  Complex cystic and solid  Hypoechoic  Isoechoic  Heterogeneous |
|  | Homogeneity | Homogeneous  Heterogeneous |  |
|  | Posterior features*** | Accentuating  Not changing  Attenuating  Shadowing | No posterior features  Enhancement  Shadowing  Combined pattern |
| Calcifications^****^ |  |  | Calcifications in a mass  Calcifications outside of a mass  Intraductal calcifications |
| Associated features  Associated findings |  | Interruption of the interface between adipose tissue and gland  Architectural distortion  Tubular structure  Coarse calcifications^****^  Echogenic foci^****^  Thickening of the Cooper ligaments  Edema  Skin thickening  Skin retraction | Architectural distortion  Duct changes  Skin changes  Skin thickening  Skin retraction  Edema  Vascularity^**^  Absent  Internal vascularity  Vessels in rim  Elasticity assessment^**^  Soft  Intermediate  High |
| Special cases |  |  | Simple cyst  Clustered microcysts  Complicated cyst  Mass in or on skin  Foreign body including implants  Lymph nodes-intramammary  Lymph nodes-axillary  Vascular abnormalities  Postsurgical fluid collection  Fat necrosis |
| Non-mass abnormalities^*^ | Distribution | Bilateral  Multiple  Scattered  Segmental  Local, Focal  Clustered, Grouped |  |
|  | Abnormality of the ducts | Duct dilatation  Duct with internal echoes  Solid echoes  Floating echoes  Linear high echoes  Echogenic foci  Duct wall thickening  Irregularity of the duct caliber |  |
|  | Hypoechoic area in the mammary gland | Patchy, Mottled  Geographic  Indistinct, Ill-defined |  |
|  | Architectural distortion | Present or Absent |  |
|  | Multiple small cysts | Present or Absent |  |
|  | Echogenic foci without a hypoechoic area | Present or Absent |  |
|  | Vascularity^**^ | Hypervascular  Hypovascular  Avascular |  |
|  | Elasticity^**^ |  |  |

* The most significant difference from the BIRADS is that the JABTS guidelines classify lesions that cannot be strictly traced as masses as "non-mass abnormalities," a category separate from masses. A study by Watanabe T et al. showed that DCIS tends to show "non-mass abnormalities" [13].

** The JABTS guidelines distinguish B-mode findings, vascularity, and elasticity, regarding them as different categories. In ACR BIRADS 2013 Ultrasound, B-mode, vascularity, and elasticity are all categorized in associated findings together.

*** In posterior features, both the terms “attenuating” and “shadowing” in the JABTS guidelines are included in “shadowing” in ACR BIRADS 2013 Ultrasound.

**** ACR BIRADS 2013 Ultrasound assesses calcification by location (calcification in a mass, outside of a mass, and intraductal calcification). In the JABTS guidelines, an obvious large calcification is categorized in a "coarse calcification," and findings that we think are microcalcifications but we cannot determine are categorized in "echogenic foci.”
